# Acquired Senescent T-Cell Phenotype Correlates with Clinical Severity in GATA Binding Protein 2-Deficient Patients

**DOI:** 10.3389/fimmu.2017.00802

**Published:** 2017-07-12

**Authors:** Raquel Ruiz-García, Carmen Rodríguez-Vigil, Francisco Manuel Marco, Fernando Gallego-Bustos, María José Castro-Panete, Laura Diez-Alonso, Carlos Muñoz-Ruiz, Jesús Ruiz-Contreras, Estela Paz-Artal, Luis Ignacio González-Granado, Luis Miguel Allende

**Affiliations:** ^1^Servicio de Inmunología, Hospital Universitario 12 de Octubre, Madrid, Spain; ^2^Instituto de Investigación I+12, Hospital Universitario 12 de Octubre, Madrid, Spain; ^3^Servicio de Hemato-Oncología Pediátrica, Hospital Universitario Miguel Servet, Zaragoza, Spain; ^4^Sección de Inmunología, Hospital General Universitario de Alicante, Alicante, Spain; ^5^Unidad de Inmunodeficiencias, Servicio de Pediatría, Hospital Universitario 12 de Octubre, Madrid, Spain; ^6^Facultad de Medicina, Universidad Complutense de Madrid, Madrid, Spain; ^7^Sección de Inmunología, Universidad de San Pablo CEU, Madrid, Spain

**Keywords:** primary immunodeficiency, GATA binding protein 2, T-cell, natural killer-cell, myelodysplastic syndrome

## Abstract

GATA binding protein 2 (GATA2) deficiency is a rare disorder of hematopoiesis, lymphatics, and immunity caused by spontaneous or autosomal dominant mutations in the *GATA2* gene. Clinical manifestations range from neutropenia, lymphedema, deafness, to severe viral and mycobacterial infections, bone marrow failure, and acute myeloid leukemia. Patients also present with monocytopenia, dendritic cell, B- and natural killer (NK)-cell deficiency. We studied the T-cell and NK-cell compartments of four GATA2-deficient patients to assess if changes in these lymphocyte populations could be correlated with clinical phenotype. Patients with more severe clinical complications demonstrated a senescent T-cell phenotype whereas patients with lower clinical score had undetectable changes relative to controls. In contrast, patients’ NK-cells demonstrated an immature/activated phenotype that did not correlate with clinical score, suggesting an intrinsic NK-cell defect. These studies will help us to determine the contribution of T- and NK-cell dysregulation to the clinical phenotype of GATA2 patients, and may help to establish the most accurate therapeutic options for these patients. Asymptomatic patients may be taken into consideration for hematopoietic stem cell transplantation when dysregulation of T-cell and NK-cell compartment is present.

## Introduction

Spontaneous or autosomal dominant heterozygous mutations in *GATA2* cause haploinsufficiency of the transcription factor GATA binding protein 2 (GATA2) (1). GATA2 is a zinc finger transcription factor essential for embryonic and definitive hematopoiesis as well as lymphatic angiogenesis (2). Germline mutations in *GATA2* predispose patients to familial myelodysplastic syndrome (MDS), acute myeloid leukemia (AML) (3), “MonoMAC” syndrome of monocytopenia with predisposition to non-tuberculous mycobacterial infection (4, 5), Emberger syndrome (1), deafness, lymphedema, and the syndrome of dendritic cells (DCs), monocytes, and B and natural killer (NK) lymphoid deficiency (“DCML deficiency”) (6).

The most common immunologic feature in GATA2-deficient patients is a B-cell lymphopenia, but with all maturation subsets present (7), they also have reduced numbers of monocytes and there are no circulating DCs. Furthermore, NK cells are diminished or partially absent with specific loss of the CD56^bright^ subset (8, 9) and T cells are elevated in percentage but sometimes with reduced absolute counts due to overall lymphopenia (7). CD4^+^ lymphocytopenia (10) with reduced numbers of naïve T cells and an accumulation of CD8^+^ TEMRA have also been observed (9).

GATA binding protein 2 haploinsufficiency is caused by many different types of mutations, ranging from non-sense (stop codons and deletions), missense (amino acid substitutions), regulatory (intronic changes leading to monoallelic expression) to large deletions (11, 12). Interestingly, there is not an absolute correlation between genotype and phenotype in GATA2 deficiency, and patients with the same mutations may exhibit different clinical features ranging from isolated neutropenia or lymphedema to MDS, AML, or severe viral infections (10).

We report four GATA2-deficient patients with different clinical phenotypes. In order to better understand the genetic, immunologic, and clinical spectrum of GATA2 disease, we have performed phenotyping and functional analysis of T and NK cells in patients with both novel and previously described mutations. We observed dysregulation in both T- and NK-cell compartments that, in the case of T cells, correlated directly with a higher clinical score. This extends our previous understanding of GATA2 deficiency by defining T-cell defects in patients with severe clinical disease.

## Materials and Methods

### Blood Samples

The study was approved by the clinical ethics committee of Hospital Universitario 12 de Octubre (Spain). Blood samples were obtained from the patients, their relatives, and healthy controls after they had given written informed consent in agreement with the principles of the Declaration of Helsinki. Patients or their parents/guardians gave written consent to publish the case reports.

### DNA Sequencing

Genomic DNA was extracted from peripheral blood samples using QIAmp DNA Mini Kit (Qiagen, Hilden, Germany). *GATA2* was directly sequenced in patients P1, P2, and P4 using specific primers and conditions described in Table S1 in Supplementary Material. The *GATA2* mutation in P3 was identified by targeted sequencing with an in-house designed panel of 192 genes involved in PID (Ampliseq, Life Technologies) and confirmed by Sanger sequencing.

### Flow Cytometry

Immunophenotyping was performed on peripheral blood for the identification of T, B, and NK cells. Intracytoplasmatic staining of cytotoxic granules in CD8 and NK cells was performed using FACSLysing and PermII buffers (BD Bioscience, Madrid, Spain). Analysis of NK-cell surface markers in GATA2-deficient patients was done in ≥200 NK cells per patient. Conjugated anti-human monoclonal antibodies are listed in Table S2 in Supplementary Material. Flow cytometry data were collected using a Beckman Coulter Navios cytometer and analyzed with Kaluza 1.5a software (Beckman Coulter, Madrid, Spain).

### NK-Cell Cytotoxicity Assays

NK-cell cytotoxic function was tested as described in Ref. (13). Briefly, peripheral blood mononuclear cells were co-cultured with 5(6)-carboxyfluorescein diacetate *N*-succinimidyl ester (CFSE) labeled K562 erythroleukemia cells at different ratios for 4 h in the presence or absence of exogenous IL-2. Dead cells were measured as CFSE+PI+K562 cells. The percentage of specific lysis was calculated in all cases according to the formula: % specific lysis = [(experimental − spontaneous)/(maximum release − spontaneous release)] × 100.

### Statistics

Statistical analysis was performed using Prism 6.0 (GraphPad). Two-tailed Student’s *t*-test with Welch’s correction when variances were significantly different was applied. The results were considered significant when *P*-values were <0.05 (*), <0.01 (**), <0.001 (***).

## Results

### Patients: Case Reports, Genetics, and Immunological Features

Patient 1 is a 12-year old female that was diagnosed in a routine work-up for neutropenia. Monocytopenia, B, and NK lymphopenia (Table 1) were highly suggestive of GATA2 deficiency. Sanger sequencing revealed a heterozygous non-sense mutation in *GATA2* (c.1009C>T; p.R337X) (1). Initial bone marrow aspirate at the age of 12 years (age of presentation) was performed and was normocellular. However, a subsequent bone marrow evaluation (age 14) showed hypoplasia and myelodysplasia (normal immunophenotype and cytogenetic analysis). Blood or platelet transfusions have not been required and she is awaiting hematopoietic stem cell transplantation (HSCT) from an unrelated donor. Despite B- and NK-cell lymphopenia, her T-cell compartment showed normal percentages of CD4 and CD8 T cells and normal absolute lymphocyte counts.

**Table 1:** Immunologic features of the patients.

Variables	Normal rangechildren	Normal range adults	P1 (12 years)	P2 (26 years)	P3 (11 years)	P4 (32 years)
Neutrophils (n°/μL)	1,800–7,600	1,800–7,400	1,800	6,600	800	6,310
Monocytes (n°/μL)	200–900	300–900	16	7	250	217
Lymphocyte (n°/μL)	1,500–4,000	1,200–3,000	3,169	679	913	2,188
**T cells**						
CD3^+^ (n°/μL)	800–2,600	850–2,250	3,106	638	880	2,168
CD4^+^ (n°/μL)	600–1,500	500–1,450	1,648	285	240	905
CD8^+^ (n°/μL)	250–1,000	160–950	1,458	319	492	1,206
DN T cells CD3^+^TCRαβ^+^CD4^−^CD8^−^ (%)	0–4	0–4	0.3	0.6	2.7	0.4
CD4^+^CD45RA^+^CD31^+^ (%)	44–60	20–44	63	31	16	NA
**T-cell proliferation**						
PHA (cpm)	>50,000	>50,000	NA	NA	45,441	NA
Anti-CD3 (cpm)	>2,000	>9,000	NA	NA	14,337	NA
PMA + ionomicin (cpm)	>60,000	>60,000	NA	NA	84,411	NA
**NK cells**						
CD3^−^CD56^+^ (n°/μL)	80–600	60–500	16	4	8	1
**NKT cells**						
CD3^+^CD56^+^ (n°/μL)	40–115	35–85	253	109	621	525
**B cells**						
CD19^+^ (n°/μL)	200–700	100–500	48	34	21	18
CD19^+^CD27^+^ (%)	7–19	11.8–34.7	36	71.3	24	62
CD19^+^IgD^+^CD27^−^ (naive) (%)	75–89	57.4–83.9	NA	15.3	50.6	25
CD19^+^IgD^+^CD27^+^ (marginal zone) (%)	2.5–7	4–11.8	NA	18.4	16.5	31.9
CD19^+^IgD^−^CD27^+^ (switching) (%)	4.5–13	6.3–24.9	NA	52.5	16.2	28.7
CD19^+^CD21 low (%)	3.3–9.5	3.36–9.53	14.3	NA	12	20.7
CD19^+^CD38^+^IgM^+^ (transitionals) (%)	1–9	1–9	NA	NA	7.8	0.5
CD19^+^CD38^+^IgM^−^ (plasmablasts) (%)	0–2.5	0–2.5	NA	NA	2.5	6
Dendritic cells CD4^+^HLA^−^DR^+^CD123^+^ (%)	0.5–1	0.5–1	0.0	0.0	0.0	0.0
**Serum immunoglobulins (mg/dL)**						
IgG (mg/dL)	600–1,230	700–1,600	1,040	2,070	643	2,580
IgA (mg/dL)	30–200	70–400	64	588	43	415
IgM (mg/dL)	50–200	40–230	73	207	98	218
**Specific antibodies**						
IgG vs pneumococcus (mg/dL)	>5.40	>5.40	NA	NA	10.4	NA
IgG2 vs pneumococcus (mg/dL)	>2.14	>2.14	NA	NA	2.88	NA
IgG vs tetanus toxoid (IU/mL)	>0.15	>0.15	0.40	NA	0.88	NA
Cytogenetics			46,XX	46,XY	+8	NA
Mutations			R337X	M236Ifs325X	K378X	T354M
			*de novo*	*de novo*	*de novo*	AD
Clinical score			0	0	2	3

Patient 2 is a 27-year old male whose clinical features were described in Ref. (14). He had suffered severe complications related to GATA2 deficiency: an extensive vesicular rash, later confirmed positive for HSV-2 by viral culture, that began on his left toe and spread to affect the entire surface of his left lower extremity, accompanied by a stable moderate lymphedema in his left leg. In addition to anemia and neutropenia, immunophenotyping revealed B- and NK-cell lymphopenia with the absence of DCs (Table 1). *GATA2* Sanger sequencing identified a novel heterozygous mutation (c.708delC) that disrupts the correct transcription of the protein leading to a premature stop codon at the amino acid 325 (p.M236Ifs325X) (14). He is currently awaiting HSCT in stable condition, receiving G-CSF twice a week and daily valacyclovir prophylaxis to minimize herpes simplex virus infection or infection recurrence.

Patient 3 is an 8-year old girl with deafness requiring a hearing aid, since age 2. She had recurrent pneumonia radiologically confirmed (at least three episodes required hospital admission). After azithromycin treatment was started respiratory infections decreased. Despite normal respiratory function tests at the age of 10, bilateral bronchiectasis and air trapping were present in a lung CT performed at that time. Physical examination revealed only mild lymphedema in her left leg. She started growth hormone therapy at the age of 9, with subsequent response to hormone replacement. She presented with lymphopenia with low naïve CD4 and CD8 T cells, impaired proliferation to PHA, elevation of TCRγδ T cells, and very low B and NK cells (Table 1). At this point, a combined immunodeficiency was suspected. ADA, RAG1, and RAG2 deficiency were excluded by Sanger sequencing. Finally, she was included in a targeted sequencing panel for PID. Gene variant analysis revealed an exonic nucleotide substitution in *GATA2* (c.1132A>T), not found in ExAC or 1,000 genomes database, that was predicted to cause a premature stop codon (p.K378X). Sanger sequencing confirmed these data. Despite normal bone marrow aspirate by optical microscopy and immunophenotyping, cytogenetic analysis revealed an abnormal karyotype 47, XX, +8 (57%, 21 cells) and hyperdiploidy of more than 80 chromosomes (24%, 21 cells) (Table 1). She underwent HSCT from a fully matched sibling donor at the age of 13.

Patient 4 is a 37-year old woman referred from the Dermatology department for evaluation of genital and axillary warts refractory to treatment, extensive vulvar intraepithelial neoplasia grade III on labia majora, and persistent lymphedema of the vulva, combined with chronic anemia and thrombocytopenia due to bone marrow failure. The clinical history of the patient included bone marrow aplasia discovered at 14 years, after an episode of typhoid fever; chronic lung disease of unknown origin, that required surgical resection of the lower lobe of the left lung for bronchiectasis at 18 years; cytomegalovirus (CMV) pneumonia at age 23 requiring ICU admission for 15 days. She currently smokes and receives chronic treatment with inhaled bronchodilators. She has a substantial family history: one sister deceased at 35 years due to disseminated infection by *Mycobacterium avium* complex; her sister also had persistent warts and suffered a blood disorder similar to the patient. Their father died at 32 years of acute leukemia (DNA samples were not available). There were no other affected family members. *GATA2* sequencing identified a nucleotide substitution (c.1061C>T) leading to p. T354M [described in Ref. (3)].

We clustered these four patients based on clinical complications to obtain the clinical score described in Ref. (9) in order to explore possible correlations between immunologic phenotype and clinical features (see Table 1; Table S3 in Supplementary Material). NK, B, and DC deficiency was observed in all four patients as well as impaired NK-cell cytotoxicity (Table 1; Figure S1 in Supplementary Material). Although B-cell numbers were globally decreased, the proportion of naïve, switched memory, and marginal zone B subpopulations were preserved.

### T-Cell Compartment Composition Exhibits Distinct Features in Accordance with Clinical Score

**Figure 1:**
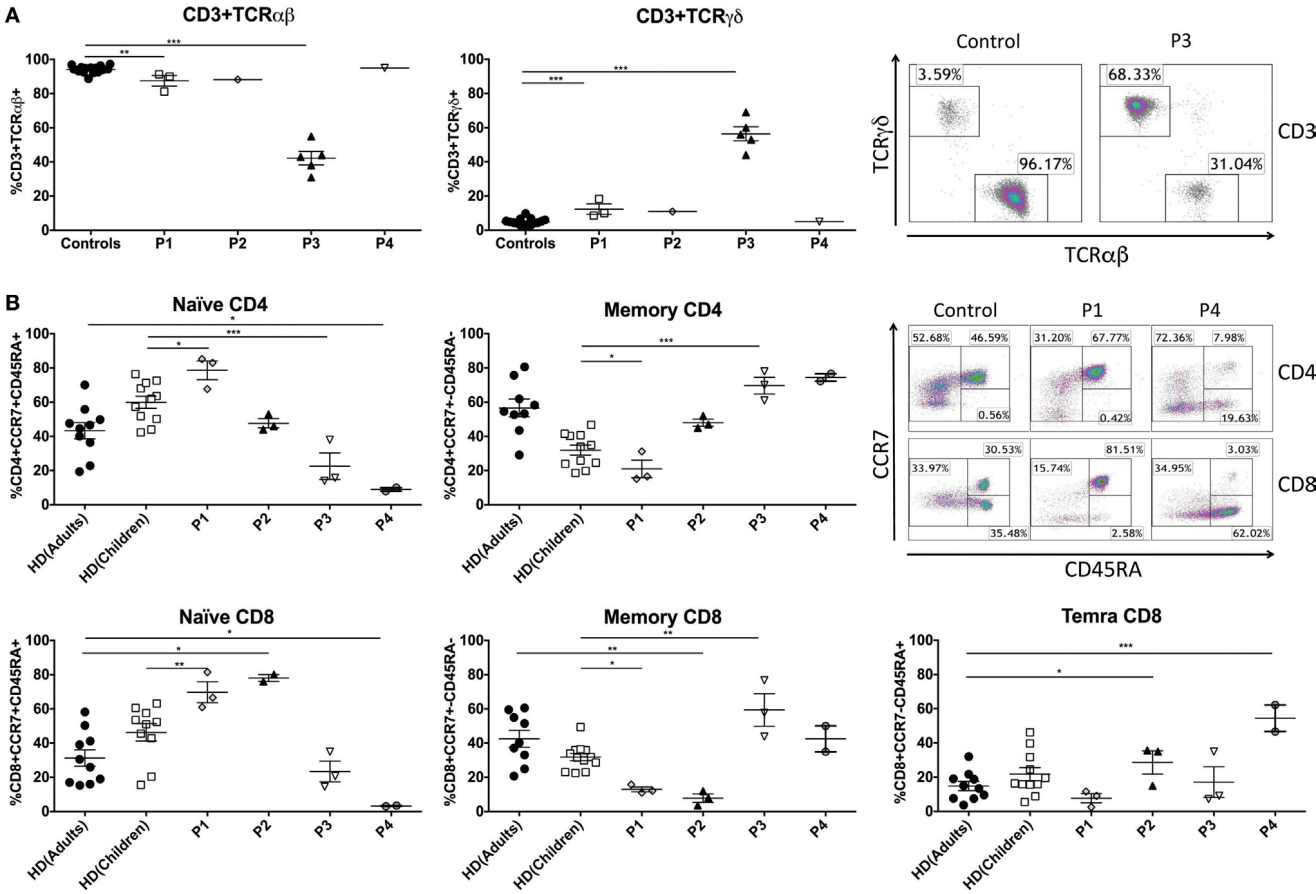
Peripheral blood T-cell compartment in GATA2-deficient patients. **(A)** Percentage of CD3^+^TCRαβ^+^ and CD3^+^TCRγδ^+^ T cells of GATA2 patients and healthy donors. Example representing CD3^+^TCRαβ^+^ and CD3^+^TCRγδ^+^ T cells from P3 and a healthy control. **(B)** CD4^+^ and CD8^+^ T cells subsets and examples of P1, P4, and a control (adult). Naïve CD4^+^ (CCR7^+^CD45RA^+^), memory CD4^+^ (CCR7^+^CD45RA^−^), naïve CD8^+^ (CCR7^+^CD45RA^+^), memory CD8^+^ (CCR7^±^CD45RA^−^), and TEMRA CD8^+^ (CCR7^−^CD45RA^+^). P1 and P3 were compared with children controls whereas P2 and P4 were compared with adult controls. Lines represent mean and bars represent the standard error of the mean. *P < 0.05; **P < 0.01; ***P < 0.001.

Abnormalities in T cells have been described in patients with GATA2 deficiency (9). Here, we wanted to assess the impact of mutations in *GATA2* on T-cell phenotypes and correlate these findings with the clinical presentations of our patients. Peripheral blood CD3^+^ T-cell numbers and TCRVβ repertoire (data not shown) were studied by flow cytometry in GATA2-deficient patients and the data were comparable with age-matched controls (Table 1). However, the ratio of CD3^+^TCRαβ^+^ and CD3^+^TCRγδ^+^ subpopulations was imbalanced in P1–3, and CD3^+^TCRγδ^+^ cells represented more than 15% of CD3^+^ T cells (controls 4.82 ± 1.99) (Figure 1A). In particular, P3 had a substantial increase in TCRγδ^+^ that reached 56.4 ± 9.1 of total CD3^+^ T cells. P4 had proportions of both subpopulations similar to those of healthy donors.

P1 and P2, the patients with fewer clinical complications and a clinical score of 0, presented normal subpopulations of CD4 and CD8 T cells compared with controls. The percentages of naïve CD4 (CD4^+^CCR7^+^CD45RA^+^), memory CD4 (CD4^+^CCR7^±^CD45RA^−^), naïve CD8 (CD8^+^CCR7^+^CD45RA^+^), memory CD8 (CD8^+^CCR7^±^CD45RA^−^), and TEMRA CD8 (CD8^+^CCR7^−^CD45RA^+^) T cells were within the range of age-matched related donors. Indeed, P1 had significantly higher levels of naïve CD4 (P1 80.66 ± 6.04%, control children 59.96 ± 11.72%) and naïve CD8 T cells (P1 66.86 ± 6.00%, control children 46.26 ± 16.16%). In contrast, P3 and P4, clinically scored as 2 and 3, respectively, exhibited a profound decrease in naïve T CD4 (P3 22.56 ± 13.39%, control children 59.96 ± 11.72%; P4 8.47 ± 2.56%, control adults 42.95 ± 15.75%) and naïve T CD8 populations (P3 23.40 ± 10.51%, control children 46.26 ± 16.16%; P4 3.25 ± 0.29%, control adults 31.33 ± 16.27%) with a corresponding increase in memory CD4 T cells in both patients (P3 69.66 ± 8.50%, control children 31.92 ± 9.75%; P4 74.79 ± 2.53%, control adults 57.12 ± 16.52%). The reduction in naïve CD8 T cells led to an elevation of memory CD8 T cells in P3 (P3 59.40 ± 15.51%, control children 31.64 ± 7.85%) whereas in P4, the patient with higher clinical score, TEMRA CD8 T cells were increased compared with controls (P4 54.43 ± 10.98%, control adults 14.83 ± 8.39%) (Figure 1B).

**Figure 2:**
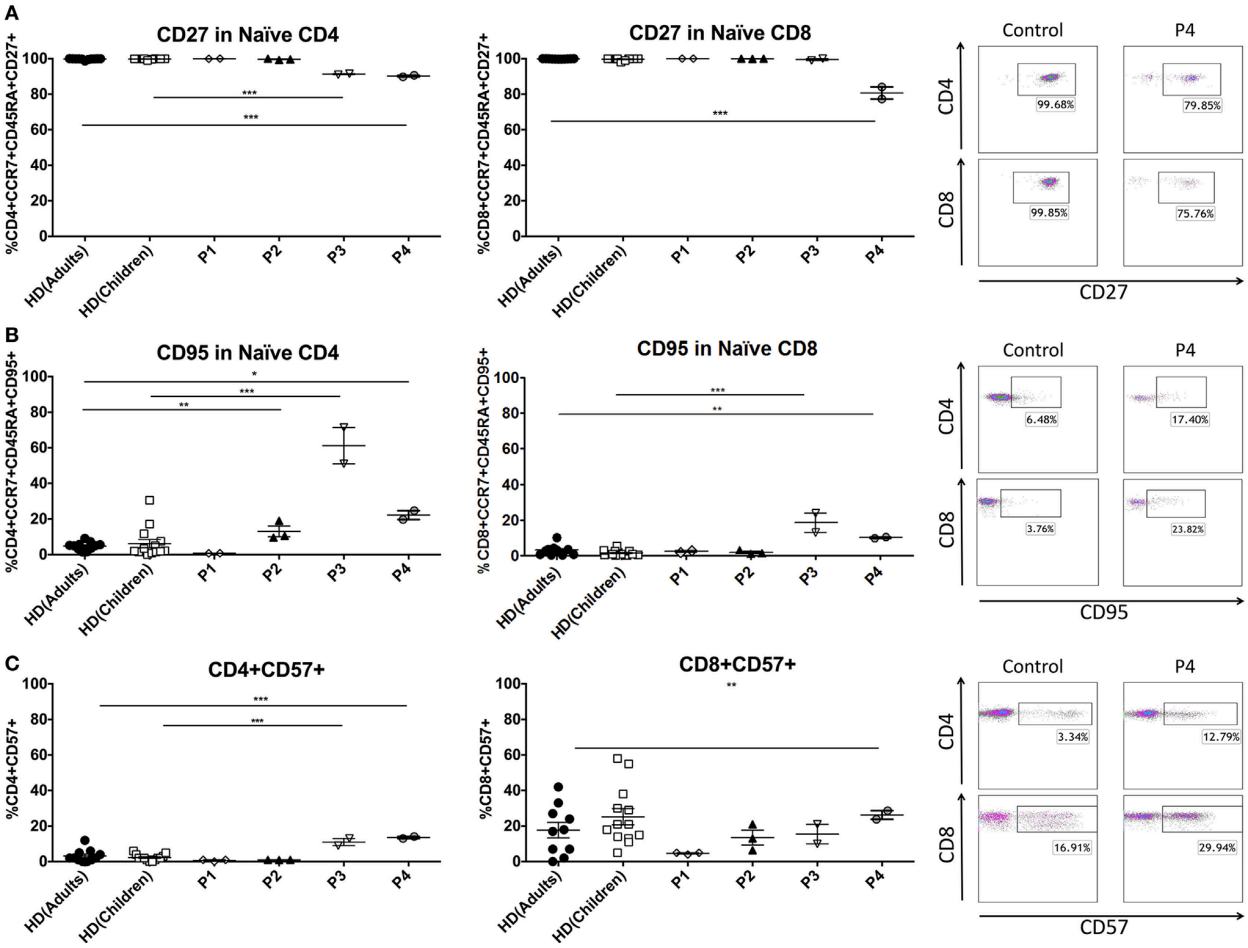
Senescence and maturation markers of peripheral T cells in GATA2-deficient patients. **(A)** CD27 expression in naïve T cells. **(B)** CD95 expression in naïve T cells. **(C)** CD57 expression in CD4 and CD8 T cells. P1 and P3 were compared with child controls whereas P2 and P4 were compared with adult controls. Lines represent mean and bars represent the standard error of the mean. *P < 0.05; **P < 0.01; ***P < 0.001.

In order to better understand the T-cell phenotype in these patients, we studied several molecules that change during differentiation from naïve to effector cells. The reduction of the naïve T-cell compartment in patients 3 and 4 was accompanied by the expression of senescence markers on the surface of these cells. Naïve T CD4 cells of P3 and P4 showed significantly increased percentages of CD95 (P3 61.21 ± 14.44%, control children 6.20 ± 8.50%; P4 22.25 ± 3.54%, control adults 4.79 ± 2.29%) with loss of CD27 (P3 91.26%, control children 99.81 ± 0.30%; P4 90.19 ± 0.61%, control adults 99.77 ± 0.38%). Similar results were found in naïve CD8 T cells, where P3 and P4 showed increased levels of CD95 (P3 18.44 ± 7.60%, control children 2.72 ± 2.42%; P4 10.04 ± 0.50%, control adults 3.01 ± 2.78%) and decreased expression of CD27 (P4 80.67 ± 4.78%, control adults 99.89 ± 0.10%). Total CD4^+^ T cells expressed significantly higher levels of CD57 in P3 and P4 (P3 11.00 ± 2.82%, control children 2.30 ± 1.79%; P4 13.57 ± 0.81%, control adults 3.18 ± 3.51%). CD57 expression was also elevated in CD8 T cells from P4 (26.24 ± 3.41%, control adults 17.70 ± 13.90%). In contrast, there was no differential expression of the CD27, CD28, CD127, CD57, and CD95 surface markers in P1 and P2 T cells (Figure 2). Intracytoplasmatic staining of cytotoxic granules in CD8 T-cell revealed reduced expression of granzyme B in P1–3, and normal values in P4. Although granzyme B expression was impaired in these patients, perforin and granzyme A staining was normal compared with healthy controls (Figure S1A in Supplementary Material). These results suggest that patients with a more severe clinical presentation of GATA2 deficiency have decreased naïve T-cell populations with a corresponding increase in terminally differentiated T cells.

### GATA2 Patients Displayed an Immature/Activated NK-Cell Phenotype and Expansion of NKT Cells

**Figure 3:**
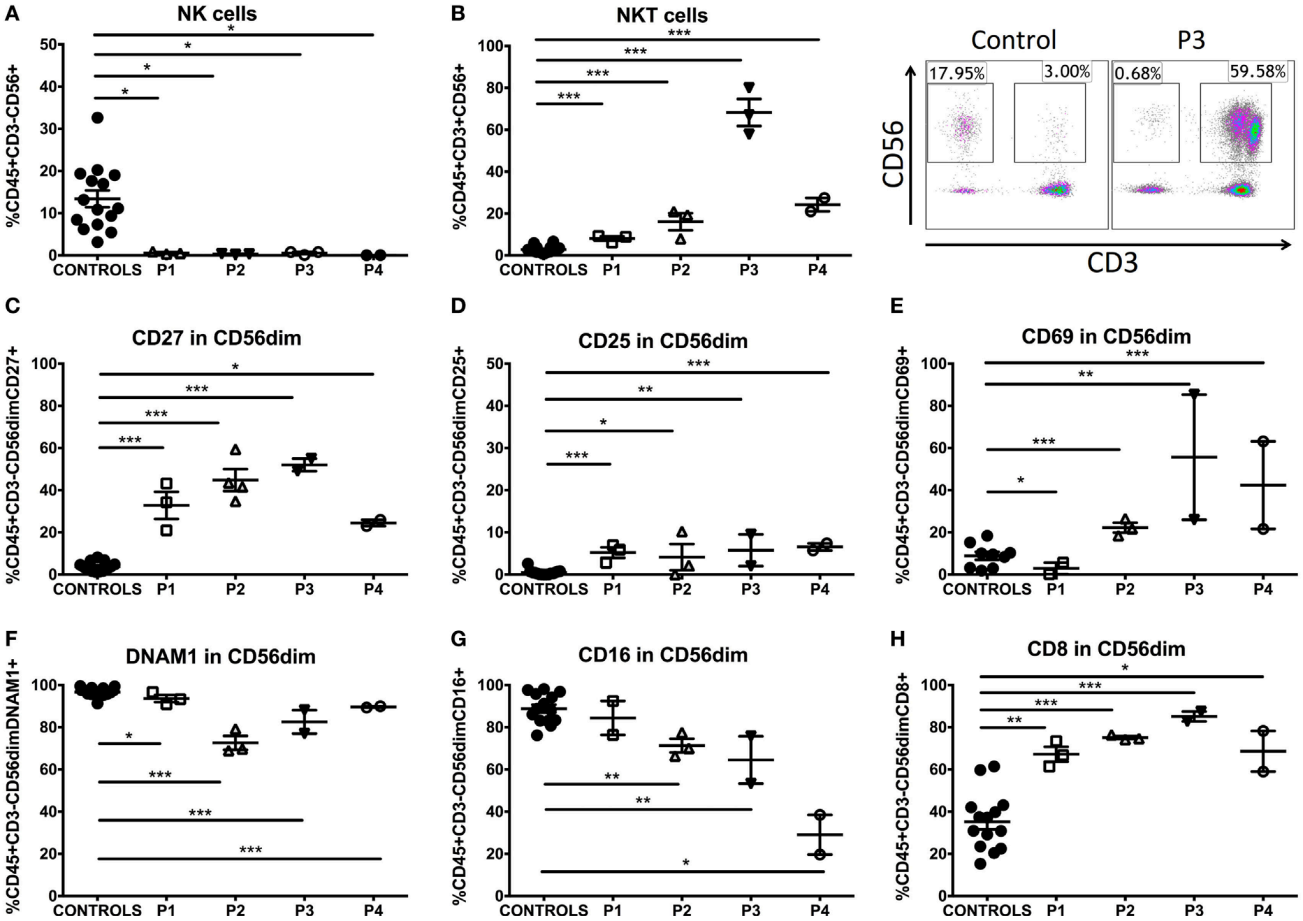
NK- and NKT-cell phenotype in GATA2-deficient patients. **(A)** Percentage of NK cells (CD3^−^CD56^+^) of GATA2 patients and controls. **(B)** Percentage of NKT cells (CD3^+^CD56^+^) of GATA2 patients and healthy donors. **(C)** CD27 expression in CD56 dim NK cells. **(D)** CD25 expression in CD56 dim NK cells. **(E)** CD69 expression in CD56 dim NK cells. **(F)** DNAM1 expression in CD56 dim NK cells. **(G)** CD16 expression in CD56 dim NK cells. **(H)** CD8 expression in CD56 dim NK cells. Lines represent mean and bars represent the standard error of the mean. *P < 0.05; **P < 0.01; ***P < 0.001.

To determine the effect of GATA2 deficiency on NK-cell function and phenotype, we performed K562 target cell lysis assays and FACS phenotyping. NK-cell cytolytic function was abolished in all four patients (Figure S1B in Supplementary Material). Moreover, percentage and absolute counts of NK cells (CD3^−^CD56^+^) were markedly decreased (Figure 3A) with a seemingly total absence of the CD56^bright^ subset as previously described (8). In addition, the NKT population (CD3^+^CD56^+^) was significantly increased in all patients (15). Patient 3 exhibited an extremely elevated percentage of NKT cells, with an NKT frequency of 68.26 ± 11.15% of total CD45^+^ lymphocytes (Figure 3B). To further study both NK- and NKT-cell phenotype in these patients, we analyzed a repertoire of 14 surface markers and 3 intracytoplasmic molecules involved in cytotoxicity (see Tables S4, S5, and Figure S2 in Supplementary Material). Our panels included markers that indicate various stages of differentiation/maturation (CD27, CD57, PERF1, GZMA, GZMB), activation/accessory receptors (CD25, CD69, CD2, CD8, CD16, DNAM1, NKG2D, NKp44, NKp46), and cell adhesion molecules (CD11a, CD11b, CD18).

We analyzed these molecules on CD56^dim^ NK cells as they are considered the mature subset of NK cells and the CD56^bright^ subset is severely decreased in GATA2 patients. We observed an impaired differentiation/maturation profile of these cells. P1–4 expressed significantly higher proportion of CD27^+^ cells than healthy controls (Figure 3C), a marker associated with immaturity of both CD56^bright^ and CD56^dim^ human NK-cell subsets (16). CD57 expression was comparable with controls in P1, P3, and P4 and slightly reduced in P2. The proportion of perforin-expressing cells was reduced in P2, P3, and P4. We did not observe differential expression of granzyme A in P1, P2, and P3, in contrast, all patients had diminished levels of granzyme B in NK cells (Figure S1C in Supplementary Material). The proportion of CD27-expressing NKT cells was elevated in P1, but there were no other findings of interest from our studies on NKT-cell differentiation/maturation markers (Table S5 in Supplementary Material). Additionally, while the percentage of positive cells was altered, there was no differential expression of these markers on a per-cell basis when MFI was measured on NK or NKT cells (data not shown).

Activation of NK cells was measured by expression of CD25 and CD69. P2–4 showed increased proportions of CD25 (Figure 3D) and CD69 expressing cells (Figure 3E), whereas P1 only had a slight increase in CD25 expression. The proportion of NK cells expressing other accessory molecules, including DNAM1, was decreased in all patients (Figure 3F). The frequency of CD16 expressing NK cells was inversely proportional to the clinical score in all patients and P4, in particular exhibited a marked decrease in CD16 expression by CD56^dim^ NK cells, while P1 presented normal levels and P2, 3 only had slight reductions (Figure 3G; Figure S1D in Supplementary Material). Higher percentages of CD8α^+^ NK cells (Figure 3H) were observed in GATA2 patient NK cells in comparison with controls while other markers shared with T cells like CD2 were expressed normally. The expression of a range of activating receptors (NKG2D, NKp44, NKp46) was similar to healthy donors (see Table S4 in Supplementary Material). NKT cells expressed higher levels of CD69 in P2–4. CD2 expression was increased in patients P1–3 (see Table S5 in Supplementary Material). Finally, there was no differential expression of cell adhesion markers such as CD18, CD11a, and CD11b compared with healthy donors in NK or NKT cells (Tables S4 and S5 in Supplementary Material).

## Discussion

The GATA2 transcription factor plays a critical role in hematopoietic stem cell (HSC) maintenance and survival (17). Its absence in Gata2^−/−^ mice causes embryonic lethality due to a deficient maturation of HSC to different hematopoietic lineages (18). In humans, haploinsufficiency of GATA2 caused by heterozygous spontaneous or autosomal dominant mutations causes a wide spectrum of clinical implications ranging from lymphedema, predisposition to mycobacterial, and viral infections to bone marrow failure with MDS/AML. Numerous mutations in *GATA2* gene have been described as the cause of this heterogeneous disease (1–4, 6, 12, 19). Here, we present a detailed analysis of four GATA2-deficient patients, two with previously reported mutations (P1 and P4) and another two where we describe two novel mutations in *GATA2*, p.M236Ifs325X and p.K378X (found in patients 2 and 3, respectively). Importantly, our analysis has revealed a correlation between the altered lymphocyte development in these patients and the different clinical pictures and clinical scores.

In GATA2 syndrome, monocytes, B, NK, and DC populations are profoundly diminished or undetectable (6) and DC were essentially absent in all four of these patients, underscoring the importance of GATA2 as an essential regulator of DC differentiation (20). Although B-cell numbers were decreased, switched memory cells were present and no apparent functional defect was detected, with all patients having correct specific responses to immunization. P2 and P3 had mild decrease of naïve CD27 B cells. Monocytes were absent in P1 and P2, suggesting that they are the two patients that best match the classic description of DCML deficiency, while P3 and P4 had close to normal monocyte numbers. In this study, three patients out of four had increased frequencies of TCRγδ T cells and all had expanded NKT-cell populations. An elevation of TCRγδ T cells has been observed in some combined immunodeficiencies such as recombination activating gene 1,2 deficiency (21, 22), CD3γ, and CD3δ (23, 24), as a consequence of CMV infection or delayed-onset combined immune deficiency with granuloma and/or autoimmunity (25–27). While the source of the defect in innate-like T cells in GATA2 deficiency is unclear, it is possible that the markedly reduced numbers of antigen presenting cells (DC, monocytes, and B cells) could produce a dysregulated increase of invariant T cells such as TCRγδ and NKT cells (9).

Abnormalities in the T-cell compartment have been described in GATA2 patients previously. Dickinson and collaborators described a cohort of patients where some showed increased frequency of terminal effector CD8^+^ T cells (28, 29). In our work, naïve T cells were decreased in number and, importantly, an abnormal phenotype of those T cells was noted in those patients with more clinical complications (P3, P4), with increased expression of CD57 and CD95 in CD4 naïve cells. In contrast, P2 had normal percentages of cells expressing these markers compared with age-matched donors and P1 even had elevated naïve CCR7^+^CD45RA^+^CD8^+^ T cells. The senescent phenotype in P3 and P4 could be associated with replicative senescence resulting from continuous antigen stimulation as seen in repeated viral infections in other primary immunodeficiencies (13, 30).

Other NK-cell immunophenotyping studies in GATA2-deficient patients have shown a largely unaffected repertoire of surface markers with some alterations in molecules like CD27 and CD117 (8). Here, NK cells of GATA2 patients showed an immature/activated phenotype, with increased proportions of CD27, CD25, and CD69 and decreased perforin and DNAM1. It is intriguing that NK cells from GATA2-deficient patients share features of both mature (CD56^dim^, Perforin, Granzyme…) and immature cells (upregulation of CD27, CD25, and CD69 and downregulation of CD16 and DNAM1). It is tempting to speculate that these NK cells could be in an intermediate maturation stage between stage 4 and stage 5 (31) in which GATA2 plays a fundamental role (32). The lack of DCs secreting IL12 and IL15 may also be an important factor contributing to the altered NK-cell maturation and activity (33, 34). With the exception of CD16 expression, the correlation between a senescent phenotype and higher clinical score that we observed for the T-cell compartment of GATA2 patients was not observed in our analysis of NK-cell phenotype.

In summary, this study expands the knowledge of GATA2 deficiency via detailed description of the different disturbances in the T- and NK-cell compartments of GATA2-deficient patients. The T-cell alterations could be secondary changes due to recurrent infections that lead to continuous antigen stimulation, but whatever the basis of these changes it is important to note that they are associated with a higher clinical score. In contrast, GATA2 deficiency produces an intrinsic NK-cell defect that seems to be independent from clinical score. With the caveat that we have studied a relatively small cohort, and it would be interesting to analyze more GATA2 patients, our data strongly suggest that the, analysis of lymphocyte subsets can provide indispensable knowledge in the symptomatic and presymptomatic stage of patients with GATA2 deficiency that could help when HSCT is being considered soon after the diagnosis.

## Ethics Statement

All subjects gave written informed consent in accordance with the Declaration of Helsinki. The protocol was approved by the “Comité de Ética de la Investigación, Hospital 12 de Octubre.” Patients or their parents/guardians gave written consent to publish the case reports.

## Author Contributions

RR-G performed the laboratory work for this study, and drafted the manuscript. CR-V, FM, CM-R, JR-C, and LG-G were responsible for the clinical management of the patients. FG-B, MC-P, LD-A, and EP-A collaborated in laboratory work. LA designed the study and drafted the manuscript. LG-G and LA contributed equally to this work.

## Conflict of Interest Statement

The authors declare that the research was conducted in the absence of any commercial or financial relationships that could be construed as a potential conflict of interest.

## Abbreviations

MDS, myelodysplastic syndrome; AML, acute myeloid leukemia; PBMCs, peripheral blood mononuclear cells; GATA2, GATA binding protein 2; VIN, vulvar intraepithelial neoplasia; CFSE, 5(6)-carboxyfluorescein diacetate N-succinimidyl ester; PI, propidium iodide; RAG1,2, recombination activating gene 1,2; CMV, cytomegalovirus; NK, natural killer.
