# Supplementary material for: Acquired Senescent T-Cell Phenotype Correlates with Clinical Severity in GATA Binding Protein 2-Deficient Patients
Source: Front Immunol. 2017 Jul 12;8:802. doi: 10.3389/fimmu.2017.00802 (PMC5506090; doi:10.3389/fimmu.2017.00802)
Supplement: Supplementary file 1 [file data_sheet_1.pdf]

SUPPLEMENTARY APPENDIX

Title: Acquired senescent T cell phenotype correlates with clinical severity in GATA2 deficient patients.

Authors: Ruiz-García R.[1,2], Rodríguez-Vigil C.[3], Marco FM.[4], Gallego-Bustos F.[1], Castro-Panete MJ.[1,2], Diez-Alonso L. [1], Muñoz-Ruiz C. [4], Ruiz-Contreras J. [2,5], Paz-Artal E.[1,2,6,7], González-Granado LI.[2,5], Allende LM.[1,2].

Supplementary Table 1. Primers used to amplify and sequence *GATA2* gene.

INTRON 5 (5'-3')	Fwd: ATCCCCGAAGTTGAGTGTCC
	Rev: AGGCTTAAATCGTTCCCTGC
EXON 3 (5'-3')	Fwd: CGGGACTGGTGCTCTTTCT
	Rev: AGGAATCGAGCTGCTGAAAA
EXON 4 (5'-3')	Fwd: CTGGTTCTGGGAGTCGTGAT
	Rev: GTCTCGCTCTAATAGCCCCC
EXON 5 (5'-3')	Fwd: AGACCCTCTCGTCCCTCTTC
	Rev: GATGTCAGTTGGGGTCGTCT
EXON 6 (5'-3')	Fwd: TCCATGGTTCTGAGATGCTTT
	Rev: AGGAGGTGGAGCTGGCTAG
EXON 7 (5'-3')	Fwd: CTATGAAGGTCGGGCACAAT
	Rev: TTACTGTGAATGTTCCCCACC

Supplementary Table 2. Monoclonal antibodies.

Antigen	Fluorochrome	Clone	Company
CCR7	FITC	150503	BD Biosciences
CD107a	PE	H4A3	BD Biosciences
CD11A	PE	25.3	Beckman Coulter
CD11B	PE	Bear1	Beckman Coulter
CD123	PE	9F5	BD Biosciences
CD127	PE	Hil-7R-M21	BD Biosciences
CD16	FITC	NKP15	BD Biosciences
CD18	FITC	L130	BD Biosciences
CD19	PE-Cy7	J4.119	Beckman Coulter
CD2	PE	S5.2	BD Biosciences
CD21	PE	B-ly4	BD Biosciences
CD25	FITC	M-A251	BD Biosciences
CD27	APC-Cy7	M-T271	BD Biosciences
CD27	PE	1A4CD27	Beckman Coulter
CD28	PE	CD28.2	BD Biosciences
CD3	APC-AF750	UCHT1	Beckman Coulter
CD3	PerCP-Cy5.5	SK7	BD Biosciences
CD38	PerCP-Cy5.5	HIT2	BD Biosciences
CD4	APC-AF750	13B8.2	Beckman Coulter
CD45	APC	2D1	BD Biosciences
CD45RA	PE-Cy7	L48	BD Biosciences
CD56	PE-Cy7	NCAM16.2	BD Biosciences
CD57	PE	NK-1	BD Biosciences
CD69	PE	TP1.55.3	Beckman Coulter
CD8	APC	SK1	BD Biosciences
CD8	FITC	SK1	BD Biosciences
CD95	FITC	DX2	BD Biosciences
CD95	PE	DX2	BD Biosciences
DNAM-1	FITC	TX25	Biolegend
GRANZIME A	PE	CB9	BD Biosciences
GRANZIME B	FITC	GB11	BD Biosciences
HLA-DR	FITC	L243	BD Biosciences
IgD	FITC	IA6-2	BD Biosciences
IgM	APC	G20-127	BD Biosciences
NKg2D	PE	1D11	Biolegend
NKp44	PE	Z231	Beckman Coulter
NKp46	PE	9E2	Biolegend
PERFORIN	FITC	δG9	BD Biosciences
TCRαβ	FITC	WT31	BD Biosciences
TCRγδ	PE	B1	BD Biosciences

Supplementary Table 3. Clinical score of patients. Described in (9).

	HPV	Mycobacterial infection	URTI	Lung	Autoimmunity	Clinical score
**P1**						0
**P2**						0
**P3**			+	+		2
**P4**	+		+	+		3

HPV (persistent infection of hands, feet, or perineum with HPV); Myco (any history of mycobacterial infection); URTI (upper respiratory tract infections, more than 3 episodes of recurrent bacterial sinusitis, otitis, or other URTI); Lung (loss of lung volume or transfer factor ,80% predicted, history of bronchiectasis, chronic bronchitis, more than one episode of pneumonia, radiologically or pathologically confirmed pulmonary alveolar proteinosis); AI (autoimmunity:
arthritis, panniculitis, or autoimmune cytopenia).

Supplementary Table 4. Surface and Intracytoplasmatic markers in CD56^{dim} NK cells

	Controls		P1			P2			P3			P4		
	mean	SD	mean	sd	*p* value	mean	sd	*p* value	mean	sd	*p* value	mean	sd	*p* value
**CD27**	3,78	2,28	32,80	11,18	<0,0005	44,80	10,41	<0,0005	51,98	4,21	<0,0005	24,47	2,07	<0,0005
**CD57**	51,95	14,98	55,24	15,00	0,739	30,03	7,83	0,032	61,25	16,78	0,437	31,50	33,23	0,147
**Perforin**	87,31	8,09	83,60	6,75	0,461	40,75	30,76	<0,0005	67,43	15,32	0,005	5,34	5,57	<0,0005
**GZMA**	64,08	20,19	76,40	-	-	45,90	38,18	0,351	64,05	40,80	0,999	9,10	7,22	0,006
**GZMB**	69,27	11,51	33,25	29,91	0,007	14,95	2,47	<0,0005	34,50	2,96	0,002	6,78	5,95	<0,0005
**CD25**	0,52	0,65	5,20	2,17	<0,0005	4,12	5,37	0,015	5,76	5,32	0,001	6,56	1,20	<0,0005
**CD69**	3,56	3,71	18,20	18,78	0,035	34,95	13,03	<0,0005	49,06	39,82	0,002	54,23	25,45	<0,0005
**CD2**	64,08	18,28	65,78	7,18	0,879	63,72	16,19	0,975	51,21	7,96	0,356	73,25	20,15	0,524
**CD8**	35,23	13,64	67,22	6,07	0,001	75,11	1,20	<0,0005	85,14	3,34	<0,0005	68,63	13,62	0,006
**CD16**	88,81	7,02	84,42	11,38	0,447	71,30	5,67	0,001	64,51	15,87	0,001	29,07	13,29	<0,0005
**DNAM1**	96,66	2,11	93,64	2,89	0,047	72,63	5,71	<0,0005	82,56	7,86	<0,0005	89,67	0,47	<0,0005
**NKg2A**	1,75	2,11	1,03	0,92	0,575	8,59	5,67	0,001	7,05	6,43	0,017	21,93	29,60	0,004
**NKg2D**	94,82	5,92	95,79	4,74	0,794	81,10	16,93	0,020	91,89	9,21	0,543	92,42	7,71	0,609
**NKp44**	1,09	1,34	2,23	0,69	0,182	2,89	2,38	0,083	1,21	0,76	0,910	35,56	36,14	<0,0005
**NKp46**	70,08	13,09	80,28	12,64	0,237	63,72	16,04	0,471	71,16	1,64	0,911	83,81	14,58	0,190
**CD11a**	99,33	0,80	99,12	1,32	0,718	96,38	6,28	0,073	97,00	4,24	0,041	98,89	1,57	0,517
**CD11b**	99,03	0,70	97,35	1,74	0,011	88,09	4,02	<0,0005	93,95	0,08	<0,0005	97,50	3,54	0,102
**CD18**	99,37	0,86	99,51	0,85	0,799	99,74	0,46	0,491	99,65	0,49	0,665	100,00	0,00	0,332

Supplementary Table 5. Surface and Intracytoplasmatic markers in NKT cells.

	Controls		P1			P2			P3			P4		
	mean	sd	mean	sd	p value	mean	sd	p value	mean	sd	p value	mean	sd	p value
**CD27**	42,49	25,62	82,91	3,16	0,019	63,41	5,75	0,133	72,72	9,62	0,132	21,51	2,11	0,282
**CD57**	47,82	23,65	57,47	18,72	0,597	53,81	14,52	0,739	61,90	12,88	0,437	63,06	5,73	0,397
**Perforin**	59,49	29,74	75,50	2,23	0,504	74,06	3,90	0,543	73,00	11,41	0,577	38,74	6,94	0,397
**GZMA**	65,15	20,47	86,32	-	-	79,25	8,49	0,409	98,37	0,30	0,082	88,80	2,91	0,184
**GZMB**	57,28	26,53	41,40	20,47	0,476	61,99	7,81	0,821	55,76	10,81	0,942	61,68	7,79	0,833
**CD25**	3,86	4,07	3,75	2,42	0,964	1,42	1,78	0,334	0,37	0,24	0,259	1,58	2,04	0,458
**CD69**	8,87	5,60	2,95	3,85	0,196	22,23	4,07	0,004	55,67	41,95	0,003	42,39	29,37	0,004
**CD2**	97,89	1,77	84,57	5,04	<0,0005	86,30	12,33	0,003	69,22	2,21	<0,0005	99,42	0,59	0,261
**CD8**	70,10	16,91	61,49	16,14	0,433	84,31	4,33	0,178	75,47	3,03	0,670	64,62	3,71	0,664
**CD16**	5,80	5,37	3,65	0,53	0,590	2,80	2,11	0,365	3,78	4,90	0,623	5,22	5,68	0,887
**DNAM1**	93,90	6,22	91,31	2,33	0,496	75,60	3,58	<0,0005	89,94	5,75	0,408	97,82	1,13	0,400
**NKg2A**	1,85	2,06	0,66	0,43	0,348	0,97	0,46	0,419	0,41	0,21	0,353	4,24	6,00	0,227
**NKg2D**	86,03	10,84	95,71	3,36	0,155	85,72	9,41	0,964	79,20	22,63	0,466	81,28	7,81	0,565
**NKp44**	1,69	2,59	1,32	0,87	0,815	0,28	0,42	0,376	1,12	0,82	0,770	6,67	8,95	0,077
**NKp46**	3,29	4,00	1,12	0,77	0,374	1,03	1,03	0,356	1,15	1,39	0,476	16,16	22,80	0,033
**CD11a**	99,40	1,09	98,15	3,08	0,216	99,91	0,13	0,436	95,00	7,07	0,018	96,63	4,63	0,040
**CD11b**	74,70	15,26	87,10	7,36	0,198	69,11	12,42	0,564	89,99	1,39	0,191	91,00	12,72	0,175
**CD18**	96,91	4,42	97,71	1,88	0,766	99,97	0,05	0,260	99,90	0,01	0,369	98,99	1,39	0,530

Supplementary Figure 1. CD8 and NK cells cytolytic molecules and NK cell cytotoxicity in GATA2 deficient patients.

(A)Percentage of perforin, granzyme A and granzyme B in CD8 T cells(B)Cytotoxicity of NK cells; the results show the percentage of K562 cells incorporating propidium iodide (PI). (C) Percentage of perforin, granzyme A and granzyme B in CD56dim NK cells. (D) Percentage of CD16+ cells in CD56dim NK cells represented in accordance to clinical score from patients. CS=0 n=2, CS=2 n=1, CS=3 n=1.Lines represent mean and bars represent the standard error of the mean (SEM). *P<.05; **P<0.01; ***P<0.001

Supplementary Figure 2. Representative dot pots of CD56^{dim} stains. CD27, CD25, CD69, DNAM1, CD16 and CD8 expression in CD45+CD3-CD56dim cells from patients and controls.
