# Supplementary figures and images for: Acquired Senescent T-Cell Phenotype Correlates with Clinical Severity in GATA Binding Protein 2-Deficient Patients
Source: Front Immunol. 2017 Jul 12;8:802. doi: 10.3389/fimmu.2017.00802 (PMC5506090; doi:10.3389/fimmu.2017.00802)

**A**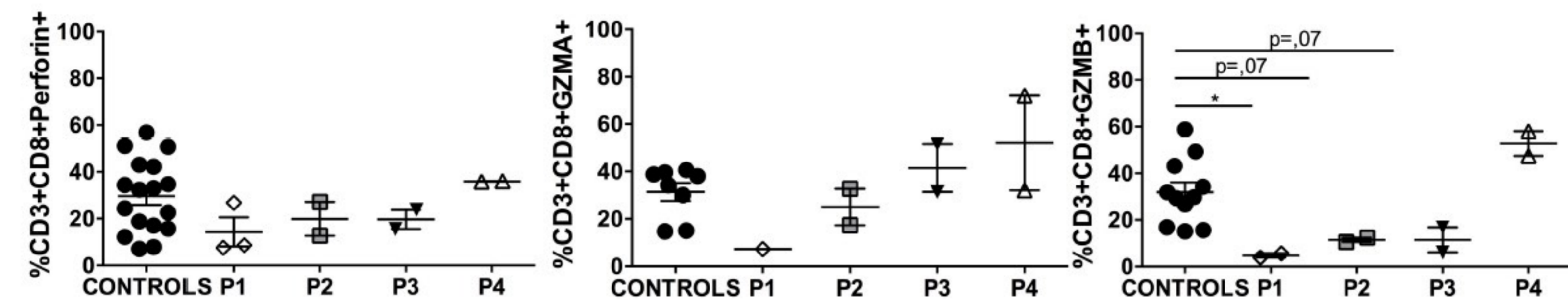**B**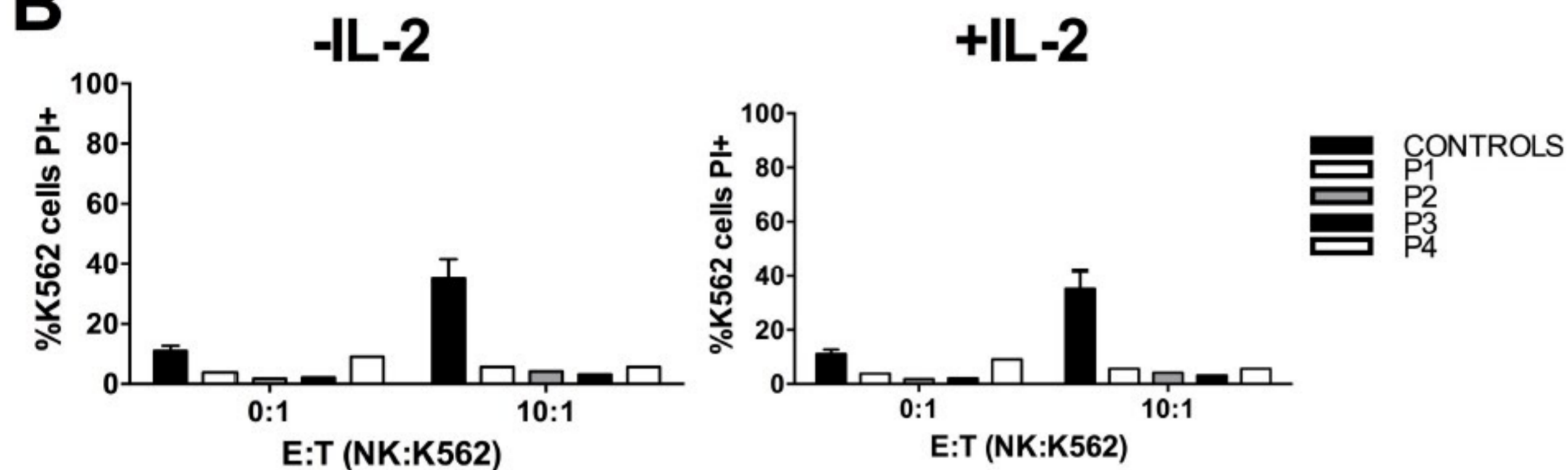**C**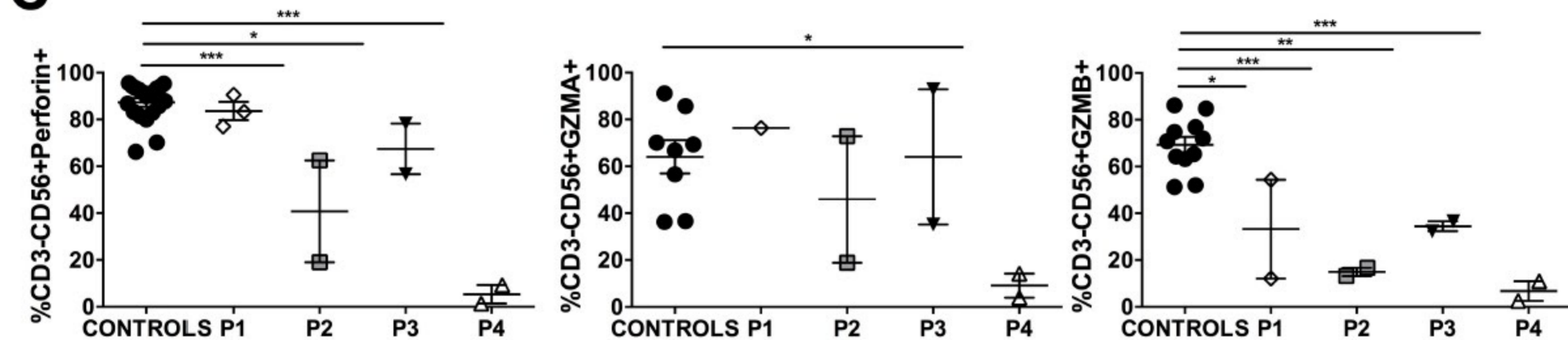**D**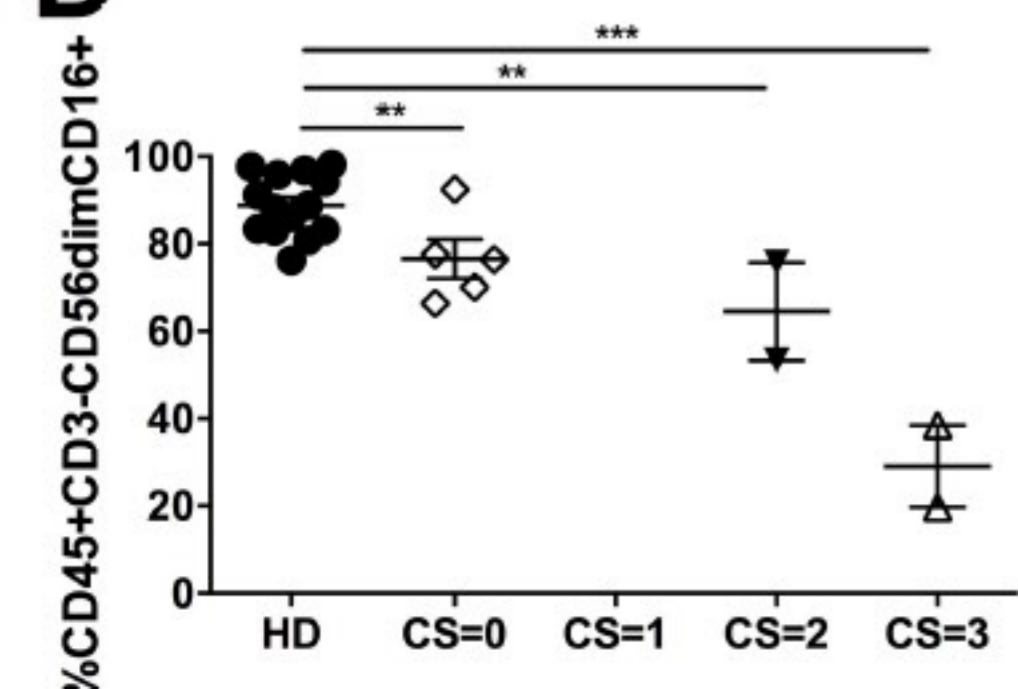

Supplement: Supplementary file 2 [file image_1.pdf]

CD45<sup>+</sup>CD3-CD56<sup>dim</sup>

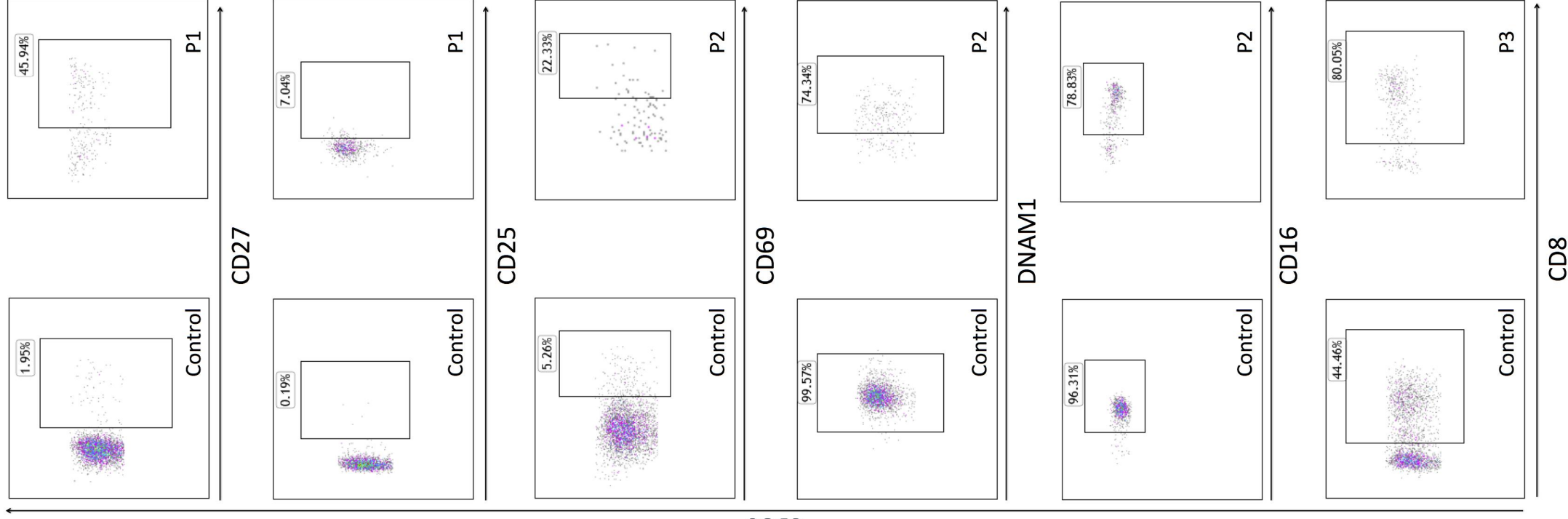

Supplement: Supplementary file 3 [file image_2.pdf]
